# Laccase-Functionalized Graphene Oxide Assemblies as Efficient Nanobiocatalysts for Oxidation Reactions

**DOI:** 10.3390/s16030287

**Published:** 2016-02-25

**Authors:** Michaela Patila, Antonios Kouloumpis, Dimitrios Gournis, Petra Rudolf, Haralambos Stamatis

**Affiliations:** 1 Biotechnology Laboratory, Department of Biological Applications and Technologies, University of Ioannina, Ioannina 45110, Greece; michaelapatila@gmail.com; 2 Department of Materials Science and Engineering, University of Ioannina, Ioannina 45110, Greece; antoniokoul@gmail.com (A.K.); dgourni@uoi.gr (D.G.); 3 Zernike Institute for Advanced Materials, University of Groningen, Nijenborgh 4, 9747 AG Groningen, The Netherlands; p.rudolf@rug.nl

**Keywords:** graphene oxide, laccase, nanoassemblies, immobilization, nanobiocatalysis

## Abstract

Multi-layer graphene oxide-enzyme nanoassemblies were prepared through the multi-point covalent immobilization of laccase from *Trametes versicolor* (TvL) on functionalized graphene oxide (fGO). The catalytic properties of the fGO-TvL nanoassemblies were found to depend on the number of the graphene oxide-enzyme layers present in the nanostructure. The fGO-TvL nanoassemblies exhibit an enhanced thermal stability at 60 °C, as demonstrated by a 4.7-fold higher activity as compared to the free enzyme. The multi-layer graphene oxide-enzyme nanoassemblies can efficiently catalyze the oxidation of anthracene, as well as the decolorization of an industrial dye, pinacyanol chloride. These materials retained almost completely their decolorization activity after five reaction cycles, proving their potential as efficient nano- biocatalysts for various applications.

## 1. Introduction

Graphene, graphene oxide and their derivatives have attracted great interest in biotechnology and biomedicine applications such as in gene and drug delivery, bioimaging, as well as in the construction of biosensors and biomedical devices [1,2,3], since they combine outstanding characteristics such as very good mechanical and thermal stability, chemical inertness, and exceptional electronic properties. More specifically, in the field of nanobiotechnology the use of graphene oxide derivatives as immobilization supports for the development of effective nanobiocatalytic systems has been intensively investigated [4,5,6,7]. The large surface area, the abundant oxygen-containing surface functionalities, and the high water solubility of these nanomaterials create an ideal immobilization support for various bioactive molecules such as genes, drugs, antibodies and other proteins, including enzymes [8,9]. There have been extensive studies on the immobilization of enzymes onto graphene oxide and their use as biosensors [10,11] and nanobiocatalytic systems in various processes [12,13].

Regarding the development of effective nanobiocatalysts through grafting of enzymes onto nanomaterials, a new interesting approach is the immobilization of enzymes in multilayer systems through layer-by-layer deposition [14,15,16]. This method can be easily applied for the development of electrochemical biosensors since the immobilization of biomolecules is achieved under biocompatible conditions, and in addition to being simple and low cost, different recognition elements can be incorporated into the layers, to construct an active material for nanodevices detecting different compounds [14]. Such multilayer assemblies have recently been shown to increase the surface molecule loading and, thus leading to a higher performance of biosensors [17,18]. Layer-by-layer deposition has been also used for the development of a reliable and reproducible fabrication scheme for solid-supported purple membrane-based devices [19], as well as for photocatalytic hydrogen production performance [20]. Furthermore, the layer-by-layer method could be also applied to realize artificial multi-enzyme complexes for *in vitro* cascade reactions [21].

In the present study, we show that functionalized graphene oxide with terminal amine groups (fGO) can be used as building block for graphene oxide-enzyme nanoassemblies put together through sequential immobilization of an oxidase (laccase) onto the fGO sheets. To the best of our knowledge, this is the first report on the development of graphene oxide-enzyme nanoassemblies by the use of a simple protocol that allows the deposition of various fGO-enzyme layers until the desired thickness of the nanostructure is achieved. The application of these laccase nanoassemblies for the degradation of polycyclic aromatic hydrocarbons and the decolorization of dyes was investigated. Laccase (*p*-diphenol:oxygen oxidoreductase E.C.1.10.3.2) is a multi-copper oxidase which catalyzes one-electron oxidation of phenolic molecules with the concomitant reduction of oxygen to water. Laccases are widely used for the development of biosensors [22,23,24,25] as well as in numerous biocatalytic applications of industrial interest [26,27,28,29,30]. These synthesized multi-layer graphene oxide-laccase nanoassemblies were found to have a very promising biocatalytic behavior (excellent oxidation activity, high operational stability, remarkable reusability). The protocol for synthesizing these novel graphene oxide-enzyme nanoassemblies should be extendable to other nanobiocatalytic assemblies with different enzyme functions and is therefore likely to have an important impact on the development of bioanalytical devices, biofuel cells and industrial biocatalysis.

## 2. Materials and Methods

### 2.1. Materials

Laccase from *Trametes versicolor* (13.6 U/mg, TvL), 2,2′-azino-bis(3-ethylbenzothiazoline-6-sulphonic acid) (ABTS), anthracene, pinacyanol chloride (1,1′-diethyl-2,2′-carbocyanine chloride) and 1-hydroxybenzoitriazole (HBT) were purchased from Fluka (St. Louis, MO, USA) and used as received. All other solvents and reagents were of HPLC or analytical grade.

### 2.2. Graphene Oxide Preparation and Functionalization

Aqueous dispersions of graphene oxide were produced using a modified Staudenmaier’s method from graphite powder (purum, ≤0.2 mm; Fluka) [31]. Graphene oxide was further functionalized using hexamethylenediamine (Sigma-Aldrich, St. Louis, Mo, USA) for the addition of amino groups, as described in previous works [32,33].

### 2.3. Immobilization of Laccase

The multipoint covalent immobilization for the synthesis of multi-layer assemblies of nanomaterial and enzyme, was achieved using glutaraldehyde (Sigma-Aldrich) as cross-linking agent between the enzyme and the amino groups located on the surface of the nanomaterial (fGO), in a similar manner as described elsewhere [34]. Briefly, 3 mg of fGO in 9.13 mL of acetate buffer (0.1 M, pH 4.58) were sonicated for 30 min in the presence of 110 μL Tween-20 (Sigma-Aldrich). After the dispersion of the nanomaterials, 1.76 mL of glutaraldehyde was added and the mixture was incubated under stirring for 1 h at 30 °C. The modified nanomaterials were separated by centrifugation at 6000 rpm and washed three times with buffer solution to remove the excess of glutaraldehyde. The nanomaterials were re-dispersed in 5 mL buffer and then 1 mL laccase solution (3 mg laccase) was added and the mixture was incubated under stirring for 1 h at 30 °C (1st layer). 2 mL of fGO (3 mg nanomaterial), previously activated with glutaraldehyde, were added to the mixture and incubated under stirring for 1 h at 30 °C (2nd layer). The above procedure was repeated till the desired number of fGO-TvL layers is achieved.

### 2.4. Determination of Immobilization Yield

The amount of immobilized enzyme was determined by calculating the protein concentration in the supernatant after the immobilization procedure using the bicinchoninic acid (BCA) assay [35]. Loading was estimated as the difference between the amount of the initial protein used and the amount of the protein in the supernatant after immobilization.

### 2.5. Atomic Force Microscopy Studies

AFM images were collected in tapping mode with a 3D Multimode Nanoscope, using Tap-300G silicon cantilevers (Ted Pella Inc., Redding, CA, USA) with a tip radius <10 nm and a force constant of ≈20–75 N·m^−1^. Samples were deposited onto silicon wafers (P/Bor, single side polished) from aqueous solutions by drop casting.

### 2.6. FT-IR Spectroscopy

Infrared spectra covering the spectral range 400–4000 cm^−1^ were measured with a FT-IR 8400 infrared spectrometer (Shimadzu, Tokyo, Japan) equipped with a deuterated triglycine sulphate (DTGS) detector. Each spectrum was the average of 128 scans collected at 2 cm^−1^ resolution. Samples were in the form of KBr pellets containing ca. 2 wt% of the material.

### 2.7. Raman Spectroscopy

Raman spectra were recorded with a Micro-Raman system RM 1000 (RENISHAW, Old Town, UK) using a laser excitation line at 532 nm (laser diode). A 0.5–1 mW laser power was used with a 1 μm focus spot in order to avoid photodecomposition of the sample.

### 2.8. Determination of the Activity of Free TvL and of fGO-TvL Nanoassemblies

Standard laccase activity was determined by oxidation of ABTS at room temperature [36]. The reaction solution was composed of 1 mM of ABTS in sodium acetate buffer (0.1 M, pH 4.58). Then a suitable amount of soluble enzyme or immobilized preparation (dispersed in acetate buffer) was added and the oxidation of ABTS was followed by measuring the absorbance change at 415 nm [36]. One unit was defined as the amount of the laccase that oxidized 1 μmol of ABTS substrate per min. All experiments were performed in triplicate.

### 2.9. Determination of the Stability of Free TvL and of fGO-TvL Nanoassemblies

Free or immobilized TvL (1 μg/mL and 50 μg/mL, respectively) was incubated up to 24 h in acetate buffer (0.1 M, pH 4.58) at 60 °C. Samples were withdrawn at predetermined time intervals to determine the remaining oxidation activity through the ABTS oxidation as described before. In the case of immobilized enzyme, the samples were first sonicated to achieve a well-dispersed mixture. All experiments were performed in triplicate.

### 2.10. Anthracene Degradation by fGO-TvL Nanoassemblies

All experiments were performed in 0.1 M sodium acetate buffer (pH 4.58) containing 1% (*v*/*v*) Tween-20, 1 mM HBT (as mediator), a final concentration of 1 mg/mL immobilized laccase and 0.1 mM concentration of anthracene in acetonitrile (Fluka). The mixtures were incubated in darkness at 30 °C under stirring at 800 rpm for 3 days. Quantitative analysis of substrates and products was performed by HPLC, using a SUPELCOSIL™ LC-C18-DB column (25 cm × 4.6 mm, 5 μm) and a diode array UV detector (Shimadzu). Linear gradient from 75% to 100% methanol (Fluka) in water (containing 0.1% acetic acid) was employed for 20 min. The elution was performed at 30 °C at a flow rate of 1 mL/min. The determination of the conversion yield was based on the decrease of the concentration of anthracene. Identification of the product (9,10-anthraquinone) was confirmed by comparison of its retention time and UV-Vis spectrum with that of the original compound.

### 2.11. Dye decolorization

The decolorization activity of fGO-TvL nanoassemblies was measured by following the color elimination of pinacyanol chloride in acetate buffer (0.1 M, pH 4.58). The reaction mixture contained 100 μg/mL pinacyanol chloride and 500 μg/mL of fGO-TvL; it was incubated at 30 °C under stirring at 800 rpm. At predetermined time intervals 20 μL aliquots were removed from the reaction mixture and added to a 1:1 (*v*/*v*) mixture of methanol and sodium acetate buffer (0.1 M, pH 4.58). The remaining concentration of the dye was monitored by measuring the absorbance at 603 nm using an extinction coefficient for pinacyanol chloride equal to ε = 82,350 M^−1^·cm^−1^ [37].

### 2.12. Reuse of fGO-TvL Nanoassemblies

The enzymatic decolorization of pinacyanol chloride was also chosen as test case in order to investigate the reusability of fGO-TvL nanoassemblies. The reaction mixture (0.5 mL) contained 100 μg/mL pinacyanol chloride in sodium acetate buffer (0.1 M, pH 4.58) and 500 μg/mL of fGO-TvL; it was incubated at 30 °C under stirring at 800 rpm for 5 h. After each reaction cycle, the fGO-TvL was separated by centrifugation at 12,000 rpm and washed three times with acetate buffer (0.1 M, pH 4.58). The fGO-TvL nanoassemblies were repeatedly used five times. The following equation was employed to estimate the decolorization yield after each reaction cycle:

$$\text{Decolorization }\left(\%\right)=\left(C_{i}-C_{t}\right)/C_{i}*100$$

where *C_i_* is the initial concentration and *C_t_* is the concentration after incubation time.

## 3. Results and Discussion

**Scheme 1:**
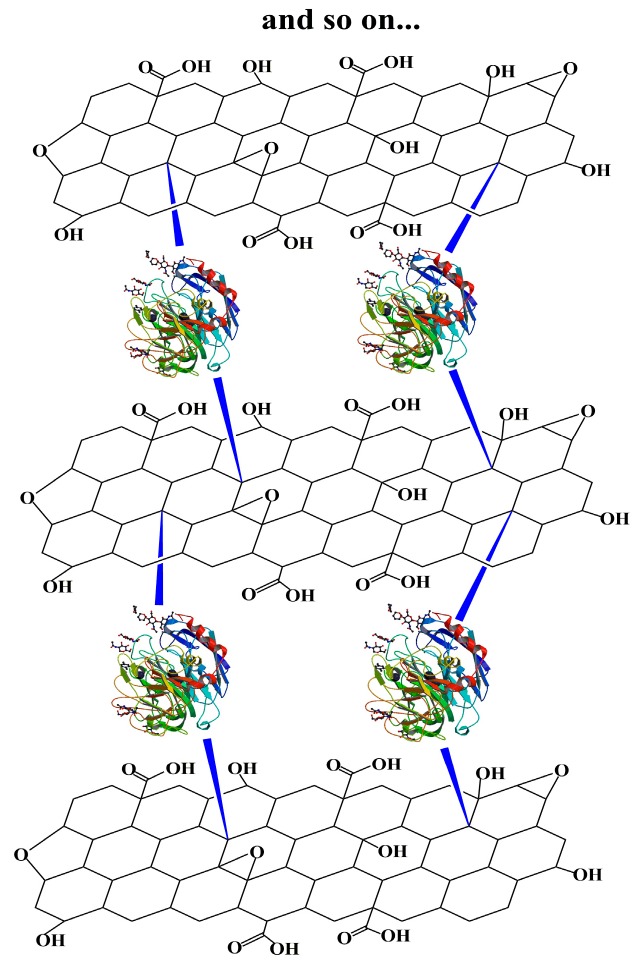
Development of graphene oxide-enzyme nanoassemblies.

In the present work nanobiocatalytic assemblies of fGO and laccase form *Trametes versicolor* (TvL) were obtained through multi-point covalent immobilization of enzyme on the surface of functionalized graphene oxide with terminal free amino-groups, using glutaraldehyde as cross-linker (Scheme 1). After grafting TvL onto the first fGO layer, the assembly was again exposed to fGO, which readily linked to the enzyme and this sequence was continued until the desired number of fGO-TvL layers was produced. As reported here below, the morphology of the nanoassemblies was probed by atomic force microscopy, their activity and thermal stability determined and their biocatalytic properties investigated using the oxidation of a polycyclic hydrocarbon (anthracene) and of an industrial dye (pinacyanol chloride) as model reactions. A reusability test concluded the study.

### 3.1. AFM

**Figure 1:**
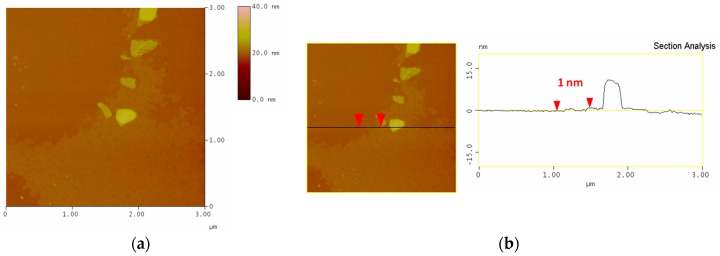
(**a**) AFM height images and (**b**) section analysis profile of a single fGO-TvL layer.

Typical AFM images of the fGO-TvL (1st layer) deposited on a Si-wafer are shown in Figure 1. The protrusions visible on top of the fGO sheet suggest that TvL has been successfully immobilized. The analysis of the topographical height profile (Figure 1b) gives an average thickness of the fGO flake of about 1 nm and of TvL of ≈11 nm.

**Figure 2:**
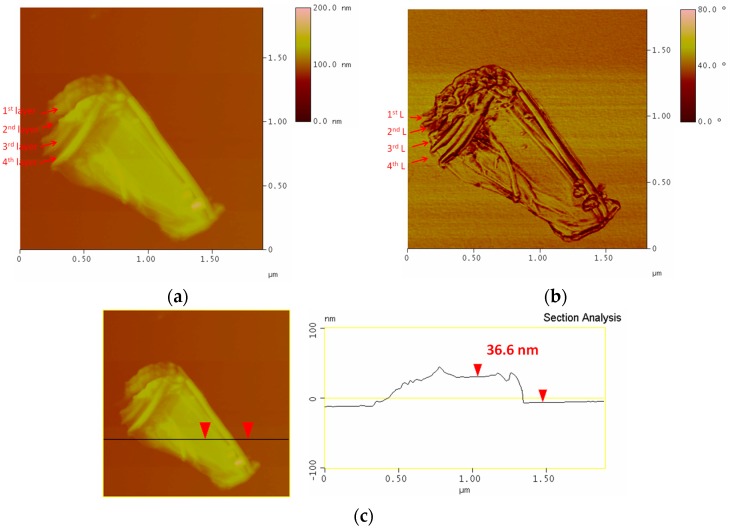
(**a**) AFM height image (**b**) phase image and (**c**) and section analysis profile of a fGO-TvL multilayer.

AFM images of a fGO-TvL multilayer (fGO-TvL-fGO-TvL-fGO-TvL-fGO) are shown in Figure 2. The multi-layer structure consisting of 4 fGO layers is visible both in height (Figure 2a) as well as in phase (Figure 2b) images. This structure was further confirmed by the topographical height profile (Figure 2c). Comparing the the overall thickness of the fGO-TvL monolayer (Figure 1b), 12 nm, with the average thickness of multilayer of circa 37 nm deduced from (Figure 2c) one concludes that the multilayer structure consists of 3 layers of fGO-TvL (3 × 12 = 36 nm) plus one functionalized graphene oxide sheet (1 nm).

### 3.2. Spectroscopic Characterization of fGO-TvL Nanoassemblies

**Figure 3:**
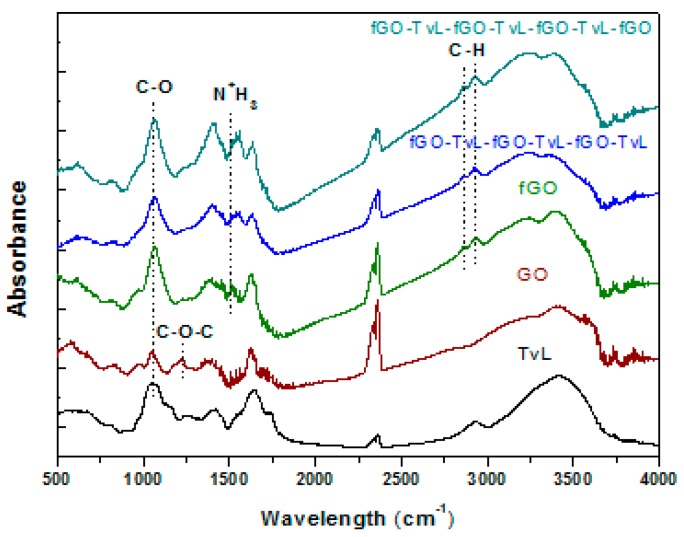
FT-IR spectra of the functionalized GO and fGO-TvL multilayer nanoassemblies compared to those of pristine GO and TvL.

The FT-IR spectra of the functionalized GO-NH_2_ and fGO-TvL multilayer nanoassemblies (fGO-TvL-fGO-TvL-fGO-TvL and fGO-TvL-fGO-TvL-fGO-TvL-fGO) were compared with those of pristine GO and TvL as shown in Figure 3. The spectra display all the characteristic bands of GO and TvL. More specifically, GO exhibits a weak band at 1620 cm^−1^ assigned to the C=O stretching vibrations of the COOH groups, a strong band at 1396 cm^−1^ assigned to the O–H deformations of the C–OH groups, a strong band at 1062 cm^−1^ attributed to C–O stretching vibrations, and a weak band at 1230 cm^−1^ assigned to asymmetric stretching of C–O–C bridges in epoxy groups and/or to deformation vibrations of O–H in the carboxylic acid groups [31]. The same bands are also present in the case of the functionalized GO and fGO-TvL multilayer nanoassemblies, while the intensity of the band at 1230 cm^−1^ decreases due to the nucleophilic substitution reactions between the amine end groups of the organic molecules and the epoxy groups of the GO [38,39], confirming the successful functionalization and immobilization of GO and enzyme, respectively. Finally, two extra bands at 2927 and 2856 cm^−1^, due to asymmetric and symmetric stretching vibrations of CH_2_ groups, confirm the presence of the amine derivatives in the fGO-TvL multilayer nanoassemblies.

**Figure 4:**
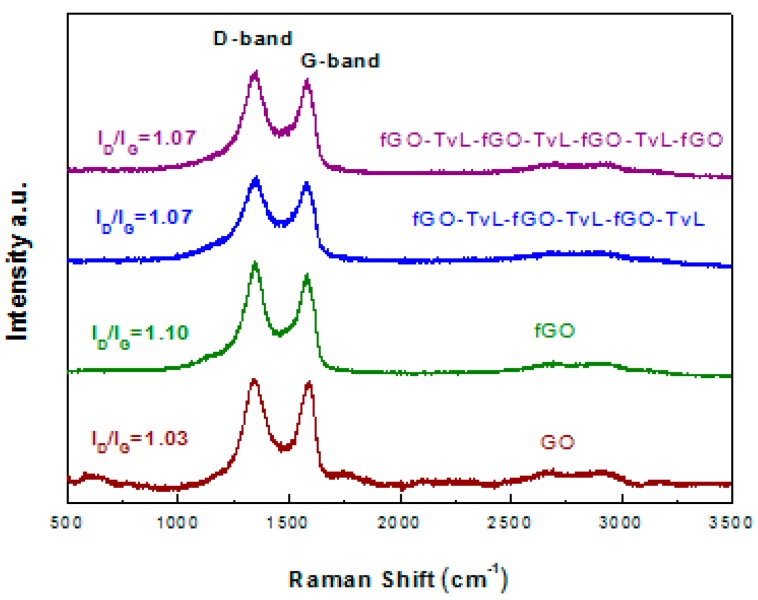
Raman spectra of the functionalized GO and fGO-TvL multilayer nanoassemblies compared to that of pristine GO.

Raman spectra of the functionalized GO and fGO-TvL multilayer nanoassemblies (fGO-TvL-fGO-TvL-fGO-TvL and fGO-TvL-fGO-TvL-fGO-TvL-fGO) compared with the pristine GO and TvL are shown in Figure 4. All spectra are similar and typical of graphene oxide-based materials without significant differences. In particular, the spectra show the characteristic G peak at 1580 cm^−1^, which is due to the first-order scattering of *E_2g_* mode, and the D band at 1349 cm^−1^, which stems from disorder in the *sp^2^*-hybridized carbon atoms and is characteristic of lattice distortions in the graphene sheets due to the extensive oxidation [40]. The D peak intensity is not related to the number of graphene layers but only to the amount of disorder. Thus, the shape and intensity ratio of D to G [I_D_/I_G_] bands can be used to evaluate the quality of graphene-based materials [41]. The I_D_/I_G_ intensity ratio was found to be 1.03 in the case of GO, while the ratios for both fGO-TvL multilayers nanoassemblies is equal to 1.07 indicating that the immobilization of the enzyme on the graphene nanosheets leaves the structure of GO unaffected.

### 3.3. Oxidation Activity of fGO-TvL Nanoassemblies

The catalytic activity of the fGO-TvL nanoassemblies was determined using the oxidation of ABTS at room temperature as model reaction. The catalytic activity of TvL was decreased from 13.6 U/mg of free enzyme to 0.55 U/mg for fGO-TvL. The reduction of the enzyme activity upon immobilization observed here is in accordance with that reported for several enzymes and could be due either to partial denaturation of enzymes during the immobilization procedure or to mass transfer effects which can lead to a reduced catalytic efficiency compared to soluble enzymes [42,43].

As it can be seen from Table 1, the catalytic activity of fGO-TvL nanoassemblies is increased as the number of fGO and enzyme layers increases. The increase of activity with the addition of a second layer of fGO is possibly due to interactions between the enzyme and the fGO sheet during the immobilization procedure that lead to a more active conformation of the enzyme. Adding a freely accesible enzyme on top of the nanoassembly, as in the case of fGO-TvL-fGO-TvL and fGO-TvL-fGO-TvL-fGO-TvL, increases the catalytic activity, which could stem from the fact that the external enzyme layer is easily accessible to substrate molecules. When instead an external layer of fGO was added on top of the enzyme, as in the case of fGO-TvL-fGO-TvL-fGO and fGO-TvL-fGO-TvL-fGO-TvL-fGO), the activity of the nanobiocatalysts decreased. This seems to indicate that as the nanoassembly gets thicker, the benefit of the extra fGO layer in inducing a more active enzyme conformation is outweighed by a reduced substrate availability when the enzyme is located in an internal layer of the nanoassembly. It is worthy to note that the addition of more than 3 fGO layers reduces the dispersion of the material in the reaction medium, which in turn could lead to a decrease of the catalytic activity as observed for fGO-TvL-fGO-TvL-fGO-Tv-fGO.

### 3.4. Stability of fGO-TvL Nanoassemblies

The thermal and operational stability of immobilized enzymes grafted onto various nanosupports is of great importance for technological applications of nanobiocatalytic systems, including biosensing and nanobiocatalysis [44,45]. In the present work, the thermal stability of the prepared fGO-TvL nanoassemblies was investigated. The remaining laccase activity of fGO-TvL nanoassemblies, was determined after incubation for up to 24 h in acetate buffer (0.1 M, pH 4.58) at 60 °C, and compared to that of the free enzyme.

**Figure 5:**
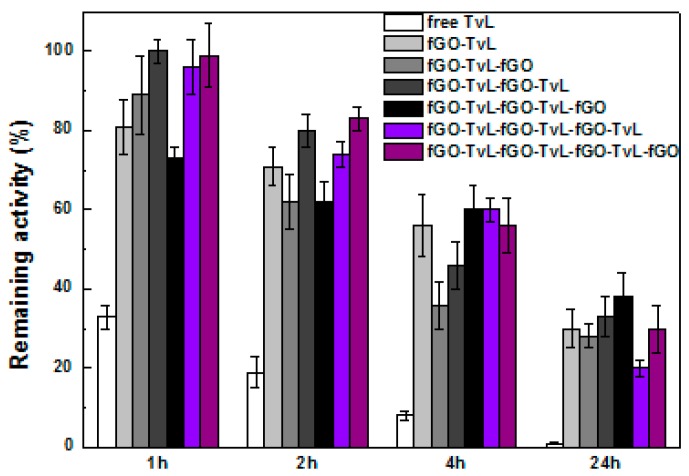
Residual catalytic activity of free TvL and of fGO-TvL assemblies after various incubation times in acetate buffer at 60 °C. 100% indicates the activity of laccase at *t* = 0 min.

Figure 5 shows the thermal stability of free and immobilized TvL, while the half-life constants of the fGO-TvL nanoassemblies are presented in Table 2. As it can be seen, all fGO-TvL nanoassemblies exhibited significant higher stability compared to the free enzyme. The free enzyme loses 90% of its initial activity after 4 h incubation at 60 °C and is almost fully deactivated after 24 h incubation. On the other hand, the multi-layer fGO-TvL nanoassemblies maintain up to 40% of their initial activity after 24 h of incubation, indicating that the immobilization of TvL onto fGO results in more stable biocatalysts compared to the native soluble enzymes, in line with literature reports on the immobilization of other enzymes on carbon-based nanomaterials [26,46,47,48]. The observed improvement in the stability of TvL may be due to the combined action of a reduced molecular mobility and an improved conformational stabilization of the enzyme resulting from the multi-point attachment of TvL to the fGO layers. In other words, the large surface area of the fGO sheets, as well as the formation of stable covalent bonds between the fGO and TvL seem to protect the conformational state of TvL, resulting in stabilization of the protein molecules against thermal denaturation. The beneficial effect of the fGO sheets for TvL stabilization increases as the number of fGO-TvL layers grows, indicating that enzyme-graphene oxide nanoassemblies can be successfully used as effective supports where enzymes are protected from thermal inactivation.

### 3.5. Use of fGO-TvL Nanoassemblies for the Oxidation of Anthracene

Polycyclic aromatic hydrocarbons (PAHs) are highly toxic industrial pollutants, resulting from processes such as coal gasification, coking, and wood preservation. Due to their toxic effects PAHs pose a serious health risk to all forms of life, including humans, and some PAHs are known to be highly mutagenic and carcinogenic, thus their degradation is of high importance [49].

In order to evaluate the ability of fGO-TvL nanoassemblies to catalyze the oxidation of PAHs, anthracene was used as model substrate and its biodegradation by fGO-TvL nanoassemblies in the presence of the mediator HBT (1 mM) was studied. The mediator HBT acts as a sort of electron shuttle between the enzyme and the substrate. Once oxidized by laccase, the mediator diffuses away from the enzymatic pocket and in turn oxidizes other molecules, extending the range of substrates susceptible to the enzymatic action and increasing the catalytic activity of the enzyme [50,51].

The degradation of anthracene by different fGO-TvL nanoassemblies is presented in Table 3; each percentage is the average of two measurements. In all cases studied, the nanoassemblies efficiently catalyze the biodegradation of anthracene, and in many cases, the conversion yield is higher compared to that of free laccase. The main intermediate oxidation product was identified as 9,10-anthraquinone, in accordance with other studies [52,53]. It is interesting to note that the degradation yield of fGO-TvL assemblies increases with the addition of fGO and enzyme layers. The degradation of anthracene by fGO-TvL (one layer) was 37.2% after 3 days of incubation at 30 °C, while the degradation efficiency of multi-layer fGO-TvL nanoassemblies under the same conditions reaches values up to 98%. These results can probably be linked to the increased stability of thicker nanoassemblies, as described above, indicating that multi-layer fGO-TvL nanoassemblies promise to be successful in applications for the detection and degradation of polyaromatic pollutants.

### 3.6. Use of fGO-TvL Nanoassemblies for Dye Decolorization

Synthetic dyes originate mainly from textile and plastic industries and are resistant to biodegradation, resulting in serious health hazards to animals and humans. Laccases have been found to be able to catalyze the oxidation of dyes and have potential applications in detection [54] and bioremediation of dye-containing industrial effluents [55].

**Figure 6:**
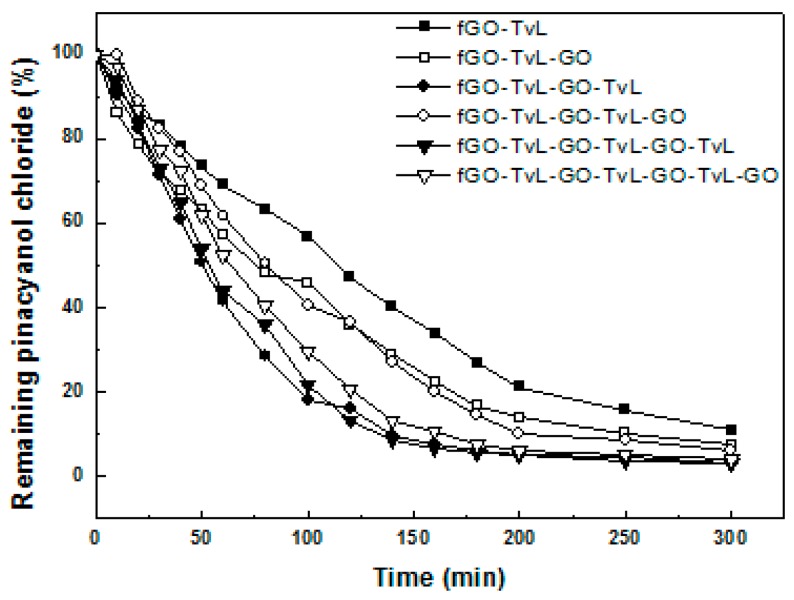
Laccase-catalyzed decolorization of pinacyanol chloride in acetate buffer 0.1 M, pH 4.58.

To investigate the ability of fGO-TvL to catalyze the degradation of dyes, we chose as model substrate pinacyanol chloride, a symmetric trimethinecyanine dye widely applied as an optical sensitizer in photographic processes [56]. Figure 6 shows the efficiency of various fGO-TvL nanoassemblies in inducing pinacyanol chloride decolorization. In all cases studied, the immobilized laccase was found to be able to decolorize the dye. The initial reaction rate for the decolorization of pinacyanol chloride catalyzed by free TvL was up to 20-fold higher than that observed for all nanoassemblies tested. However, we would like to note that the low operational stability observed for free TvL (Table 2), as well as the fact that the free enzyme cannot be reused, make the application of free enzyme for such a process inefficient in an industrial setting. As seen in Table 4, the reaction rate for the decoloriztion of pinacyanol chloride catalyzed by multilayered nanoassemblies such as fGO-TvL-fGO-TvL-fGO-TvL is up to 2.2 times higher compared to that observed for a single layer of immobilized laccase (fGO-TvL). The reaction rate of these multilayered nanoassemblies slightly decreases when an external layer of fGO was added (as in the case of fGO-TvL-fGO-TvL-fGO and fGO-TvL-fGO-TvL-fGO-TvL-fGO), which is probably correlated to reduced substrate availability as discussed above for the oxidation of ABTS by fGO-TvL nanoassemblies.

### 3.7. Reusability of the Nano-Ezyme Assemblies

**Figure 7:**
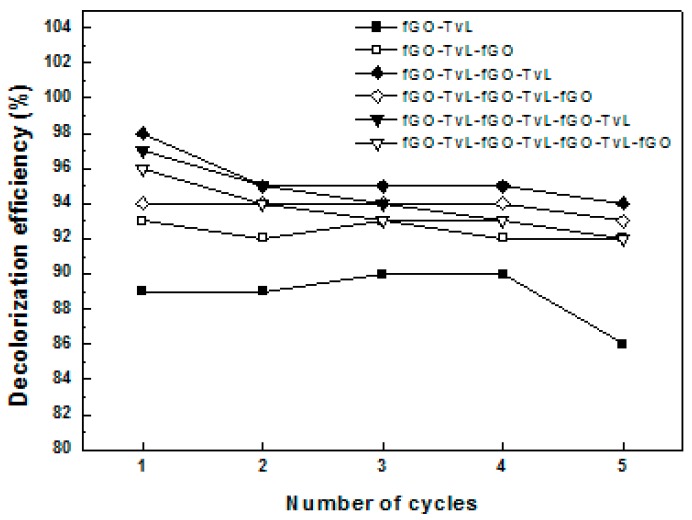
Reusability of fGO-TvL nanoassemblies in the batch oxidation of pinacyanol chloride.

If fGO-TvL nanoassemblies are to be employed for sustainable industrial applications, waste minimisation by reuse is an important issue. Reuse was therefore investigated with the oxidation of pinacyanol chloride as model reaction. As it can be seen from Figure 7, when five reaction cycles (25 h of total operation) were completed, the ability of graphene oxide-enzyme nanoassemblies to catalyzed the decolorization of dye was highly maintained (up to 94%), especially when multi-layer fGO-TvL nanoassemblies were used as biocatalyst. This indicates that the addition of extra fGO-TvL layers to the nanoassembly not only benefits the thermal stability and reaction efficiency, but also results in reusable nanobiocatalysts.

## 4. Conclusions

In the present study, we demonstrated the development of novel graphene oxide-enzyme assemblies through the multi-point covalent immobilization of laccase from *Trametes versicolor* on functionalized graphene oxide and their use in different biocatalytic applications. The catalytic activity of the fGO-TvL nanoassemblies, as well as their ability to catalyze the degradation of anthracene and pinacyanol chloride, increased with the number of nanomaterial and enzyme layers and was higher when the external layer of the multi-layer nanoassemblies was formed by the enzyme. Moreover, the graphene oxide-enzyme nanoassemblies exhibited higher thermal stability than the free enzyme and reactivity was maintained at higher temperatures when the number of fGO-TvL layers increased. The high catalytic activity against various substrates together with the enhanced thermal and operational stability, make these structures promising nanobiocatalytic systems for use in bioanalytical devices, biofuel cells and biotransformations.

## Figures and Tables

**Table 1:** Activity of fGO-TvL nanoassemblies through ABTS oxidation.

Immobilized TvL	Activity (U/mg of Immobilized Enzyme)
fGO-TvL	0.55 ± 0.07
fGO-TvL-fGO	1.00 ± 0.13
fGO-TvL-fGO-TvL	1.63 ± 0.15
fGO-TvL-fGO-TvL-fGO	0.97 ± 0.10
fGO-TvL-fGO-TvL-fGO-TvL	4.89 ± 0.63
fGO-TvL-fGO-TvL-fGO-Tv-fGO	1.05 ± 0.17

**Table 2:** Half-life constants of fGO-TvL nanoassemblies.

Enzyme	*t*_1/2_ (h)
Free TvL	0.9 ± 0.1
fGO-TvL	3.7 ± 0.4
fGO-TvL-fGO	2.9 ± 0.2
fGO-TvL-fGO-TvL	2.3 ± 0.1
fGO-TvL-fGO-TvL-fGO	4.1 ± 0.5
fGO-TvL-fGO-TvL-fGO-TvL	4.3 ±0.4
fGO-TvL-fGO-TvL-fGO-TvL-fGO	4.1 ± 0.5

**Table 3:** Degradation of anthracene by fGO-TvL nanoassemblies.

Enzyme	Anthracene Degradation (%)
Free TvL	96.5
fGO-TvL	37.2
fGO-TvL-fGO	89.3
fGO-TvL-fGO-TvL	98.6
fGO-TvL-fGO-TvL-fGO	93.8
fGO-TvL-fGO-TvL-fGO-TvL	97.4
fGO-TvL-fGO-TvL-fGO-TvL-fGO	97.8

**Table 4:** Reaction rates of fGO-TvL nanoassemblies for the decolorization of pinacyanol chloride.

Immobilized TvL	Reaction Rate (μM·min^−1^·μg^−1^ Enzyme)
fGO-TvL	0.20 ± 0.03
fGO-TvL-fGO	0.36 ± 0.05
fGO-TvL-fGO-TvL	0.45 ± 0.07
fGO-TvL-fGO-TvL-fGO	0.38 ± 0.06
fGO-TvL-fGO-TvL-fGO-TvL	0.46 ± 0.08
fGO-TvL-fGO-TvL-fGO-TvL-fGO	0.43 ± 0.08

## Abbreviations

The following abbreviations are used in this manuscript:

ABTS: 2,2′-azino-bis(3-ethylbenzothiazoline-6-sulphonic acid
AFM: Atomic Force Microscopy
fGO: Functionalized graphene oxide with terminal amine groups
HBT: 1-hydroxybenzoitriazole
HPLC: High-Performance Liquid Chromatography
PAHs: Polycyclic aromatic hydrocarbons
TvL: Laccase from *Trametes versicolor*
UV-Vis: Ultra-violet-Visible
